# Green Synthesis and the Evaluation of a Functional Amphiphilic Block Copolymer as a Micellar Curcumin Delivery System

**DOI:** 10.3390/ijms241310588

**Published:** 2023-06-24

**Authors:** Radostina Kalinova, Georgy Grancharov, Jordan Doumanov, Kirilka Mladenova, Svetla Petrova, Ivaylo Dimitrov

**Affiliations:** 1 Institute of Polymers, Bulgarian Academy of Sciences, Akad. G. Bonchev St., bl. 103-A, 1113 Sofia, Bulgaria; kalinova@polymer.bas.bg (R.K.); granchar@polymer.bas.bg (G.G.); 2 Department of Biochemistry, Faculty of Biology, Sofia University “St. Kliment Ohridski”, 8 Dragan Tzankov Blvd., 1164 Sofia, Bulgaria; doumanov@biofac.uni-sofia.bg (J.D.); k_mladenova@biofac.uni-sofia.bg (K.M.); spetrova@biofac.uni-sofia.bg (S.P.)

**Keywords:** solvent-free polymerization, amphiphilic copolymers, functional polymer micelles, nanocarriers, drug delivery, curcumin, stability, antioxidant activity, cytotoxicity

## Abstract

Polymer micelles represent one of the most attractive drug delivery systems due to their design flexibility based on a variety of macromolecular synthetic methods. The environmentally safe chemistry in which the use or generation of hazardous materials is minimized has an increasing impact on polymer-based drug delivery nanosystems. In this work, a solvent-free green synthetic procedure was applied for the preparation of an amphiphilic diblock copolymer consisting of biodegradable hydrophobic poly(acetylene-functional carbonate) and biocompatible hydrophilic polyethylene glycol (PEG) blocks. The cyclic functional carbonate monomer 5-methyl-5-propargyloxycarbonyl-1,3-dioxane-2-one (MPC) was polymerized in bulk using methoxy PEG-5K as a macroinitiator by applying the metal-free organocatalyzed controlled ring-opening polymerization at a relatively low temperature of 60 °C. The functional amphiphilic block copolymer self-associated in aqueous media into stable micelles with an average diameter of 44 nm. The copolymer micelles were physico-chemically characterized and loaded with the plant-derived anticancer drug curcumin. Preliminary in vitro evaluations indicate that the functional copolymer micelles are non-toxic and promising candidates for further investigation as nanocarriers for biomedical applications.

## 1. Introduction

According to the general requirements of green chemical processes, the applied technique should avoid the usage of organic solvents and should enable achieving quantitative conversions without any by-products that need to be removed [1]. Under certain conditions, the bulk ring-opening polymerization of cyclic monomers can be considered a green process [2]. Thus, functional polymers suitable for various applications could be obtained using specifically designed cyclic monomers [3,4,5]. Among others, the biomedical applications of such polymers are of particular interest [6,7,8].

Due to their excellent biocompatibility, low toxicity, enhanced blood circulation time, and ability to solubilize a variety of hydrophobic drugs in their core, polymeric micelles formed via the self-assembly of amphiphilic copolymers in aqueous media have been attracting enormous attention as smart drug and gene delivery vehicles [9]. Moreover, the versatility of available monomers that can be combined in different polymer architectures bearing desired functionalities enables the fine-tuning of the micelles’ properties [10]. Thus, a high accumulation and cellular uptake of the drug-loaded nanovehicles via the so-called enhanced permeability and retention (EPR) effect (passive targeting) or selectively targeted diseased cells and tissues (active targeting) could be achieved [11,12,13]. However, drug delivery using polymer nanocarriers may also face challenges, such as the presence of some toxic material used during the synthetic procedures. Therefore, it is important for the polymerization process to rely as much as possible on green technologies. Typically, numerous hydrophilic and biocompatible polymers such as poly((2-dimethylamino)ethyl methacrylate) (PDMAEMA) [14], poly(*N*-vinyl-2-pyrrolidone) (PVP) [15], poly(2-hydroxyethyl methacrylate) (PHEMA) [16], poly(2-oxazoline)s [17], poly(acrylic acid) (PAA) [18], or poly(amino acids) [19] are used as corona-forming segments of the block copolymer micelles for drug delivery applications. However, the most widely used nonionic hydrophilic polymer is poly(ethylene glycol) (PEG), which is characterized by excellent biocompatibility and antifouling properties [20]. PEG is approved by the US Food and Drug Administration (USFDA) for application in the human body and enables prolonged blood circulation of the corresponding nanocarriers due to the so-called “stealth” effect, leading to a minimal risk of particles’ opsonization followed by their elimination via reticuloendothelial system uptake [21,22]. Additionally, it has been demonstrated that PEG can be end-modified with suitable functionalities, thus imparting active targeting properties of the copolymer micelles [23]. The hydrophobic blocks of amphiphilic copolymers form the core of the corresponding micelles in aqueous media, and they are capable of solubilizing and protecting the drug during systemic circulation. The delicate balance between the micelles’ drug-loading efficiency and the following release into the target site is governed by the drug–hydrophobic polymer interactions and the structural parameters of the block copolymers [24]. This balance can be finely tuned through an appropriate choice of hydrophobic segments for the amphiphilic copolymers. Commonly used hydrophobic polymers are biodegradable polyesters such as polylactide (PLA) or poly(ε-caprolactone) (PCL), polyoxazoline-based polymers, and protected hydrophobic poly(amino acids) [25]. Additionally, aliphatic polycarbonates also show great potential for biomedical applications since they exhibit a unique combination of biodegradability and biocompatibility [26]. Their synthesis can be relatively easily achieved by the ring-opening polymerization (ROP) of corresponding cyclic carbonates [27]. Amphiphilic block copolymers of various architectures containing carbonate units in their hydrophobic segments have been synthesized and evaluated as drug-delivery systems [28,29]. The introduction of various functionalities along the hydrophobic polycarbonate segments of the amphiphilic block copolymers gives an additional option to tune the micelles’ core properties either via enhancing drug solubilization or external stimuli-triggered core disintegration and subsequent drug release [30,31,32].

Curcumin (Curc) is a natural polyphenolic substance representing the main active component isolated from the roots of the plant *Curcuma longa* L. Curc has attracted enormous interest due to its numerous biological activities such as antioxidant, anticarcinogenic, antimicrobial, anti-inflammatory, hypoglycemic, hepato- and neuroprotective, etc. [33]. However, the poor solubility, rapid metabolism, and rapid elimination lead to a limited bioavailability of Curc. In order to overcome this issue, curcumin has been encapsulated in various nanocarriers such as lipid-based nanoparticles, carbon-based nanostructures, silica nanoparticles, polymeric micelles, and dendrimers [34,35,36,37]. The drug’s efficiency was significantly increased by using multifunctional polymeric micelles as carriers [38,39].

Herein, we present the solvent-free green synthesis of the functional polycarbonate-based amphiphilic block copolymer. A monohydroxy PEG was used as a macroinitiator for the metal-free bulk ring-opening polymerization of an alkyne-functional cyclic carbonate under relatively mild conditions. The block copolymer was characterized, and its self-assembly in aqueous media was evaluated. Stable spherical micelles with an average diameter of 44 nm were formed and were physico-chemically characterized. The polymer micelles were successfully loaded with the natural drug curcumin, and parameters such as drug loading efficiency and drug loading capacity were estimated. The drug release profiles in different media were studied. Initial in vitro evaluations for nanocarriers’ stability in various simulated biological media, as well as the antioxidant activity assay of micellar curcumin, were also performed. Finally, the in vitro cytotoxicity of the empty and drug-loaded nanocarriers was evaluated.

## 2. Results

### 2.1. Solvent-Free Green Synthesis and Characterization of a Functional Amphiphilic Poly(ethylene glycol)-block-polycarbonate (MPEG-b-PC) Diblock Copolymer

**Scheme 1:**
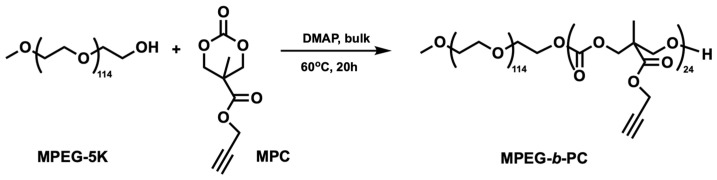
The solvent-free synthetic route to a functional amphiphilic poly(ethylene glycol)-*b*-polycarbonate diblock copolymer.

One of the key aspects of green chemistry is the removal of solvents in synthetic processes or the replacement of toxic solvents with environmentally benign ones. Accordingly, a solvent-free polymerization route to a polycarbonate-based amphiphilic diblock copolymer intended for biomedical applications is presented in Scheme 1.

**Figure 1:**
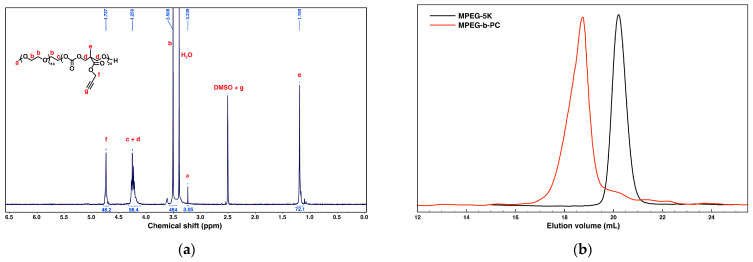
Functional amphiphilic block copolymer (MPEG-*b*-PC) characterization via (**a**) ^1^H NMR (600 MHz) spectroscopy in DMSO-d_6_; (**b**) GPC in tetrahydrofuran vs. polystyrene narrow molar mass standards.

Well-defined methoxy-poly(ethylene glycol) with narrow dispersity (*Ð*_M_ 1.03) was used as a macroinitiator for the ring-opening polymerization of an alkyne-functional cyclic carbonate monomer. The polymerization was performed in bulk at the moderate temperature of 60 °C in an inert atmosphere. Under these conditions, the macroinitiator melted and served as a reaction medium solubilizing the monomer. Moreover, potential side reactions, such as polycarbonate homopolymer formation that might occur at elevated temperatures (above 100 °C), were avoided. Keeping in mind the intended biomedical application of the diblock copolymer the metal-free organic catalyst, 4-dimethylaminopyridine (DMAP) was chosen for the current synthetic procedure. Controlled organocatalyzed ring-opening polymerization was initially reported for the metal-free synthesis of the polyesters and was further extended to the polymerization of cyclic carbonates [40,41]. The solvent-free polymerization of the alkyne-functional cyclic carbonate proceeded for 20 h, and the crude product was purified in 2-propanol in order to remove the potentially unreacted macroinitiator and the catalyst. The isolated block copolymer was analyzed to determine the molar mass characteristics. With the knowledge of the macroinitiator’s molar mass, the average degree of functional cyclic carbonate polymerization (*DP_n_*) was estimated by ^1^H NMR spectroscopy in DMSO-d_6_ (Figure 1a). By comparing the integral intensities of oxyethylene protons from the macroinitiator at 3.50 ppm and the methylene protons located next to the terminal alkyne group at 4.73 ppm, the average degree of MPC polymerization was calculated to be 24 (Figure 1a). It should be noted that the polymerization process under the chosen conditions is highly efficient with practically complete monomer conversion and high macroinitiator efficiency. The target degree of carbonate polymerization calculated from the molar ratio between the monomer and the macroinitiator in the feed (t-*DP_n_*) was 25.

The formation of diblock polymer architecture was confirmed by GPC analysis (Figure 1b). There is a clear shift toward higher molar masses of the diblock copolymer compared to the macroinitiator. The molar mass distribution of the diblock copolymer remains monomodal with a somewhat increased but still narrow dispersity. The estimated dispersity of the solvent-free synthesized amphiphilic block copolymer (*Ð*_M_ = 1.24) is slightly higher than the block copolymers with similar composition obtained in an organic solution (*Ð*_M_ = 1.05–1.12) [42]. The result is not surprising for the polymerization process performed in bulk. However, the slightly increased block copolymer dispersity is a reasonable compromise taking into account the complete removal of the organic solvents from the synthetic procedure.

**Figure 2:**
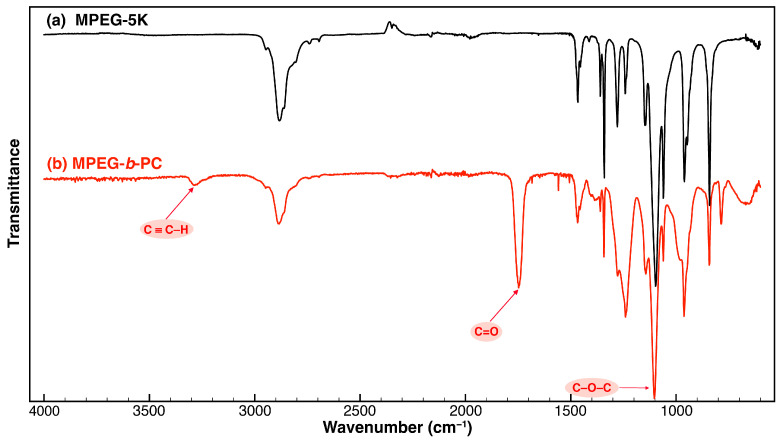
FTIR spectra of (**a**) MPEG-5K macroinitiator; (**b**) the corresponding functional amphiphilic diblock copolymer (MPEG-*b*-PC).

The successful polymerization was also evidenced by FTIR analysis (Figure 2). The strong band at 1100 cm^−1^ was attributed to the stretching vibrations of the C-O-C groups from the polyether chain in the macroinitiator’s spectrum (Figure 2a). An additional strong band at 1748 cm^−1^ corresponding to C=O stretching vibrations appeared in the FTIR spectrum of the block copolymer and was attributed to the polycarbonate block (Figure 2b). Moreover, the band at 3287 cm^−1^ is characteristic of stretching vibrations of C≡C–H from the alkyne side groups of the newly formed polymer block.

The molar mass characteristics of the macroinitiator and the functional diblock copolymer are summarized in Table 1.

### 2.2. Self-Assembly and Micelles Physico-Chemical Characterization

The amphiphilic nature of the synthesized block copolymer is a prerequisite to its self-assembly in aqueous media driven by the hydrophobic interactions between the polycarbonate units in order to minimize energetically unfavorable hydrophobe–water interactions, thus forming the core of the micelle, whereas the structure would be stabilized by a shell of the hydrophilic PEG chains. Furthermore, the core of the micelle would be functionalized with numerous alkyne “clickable” groups allowing for further modifications. The block copolymer self-assembly capability was evaluated by applying the nanoprecipitation technique. Initially, the copolymer was dissolved in acetone, which is a good solvent for both blocks followed by a dropwise addition to vigorously stirred water, which is a strong selective solvent for PEG. After the organic solvent evaporation and concentration adjustment, the aqueous dispersion was subjected to further analyses.

**Figure 3:**
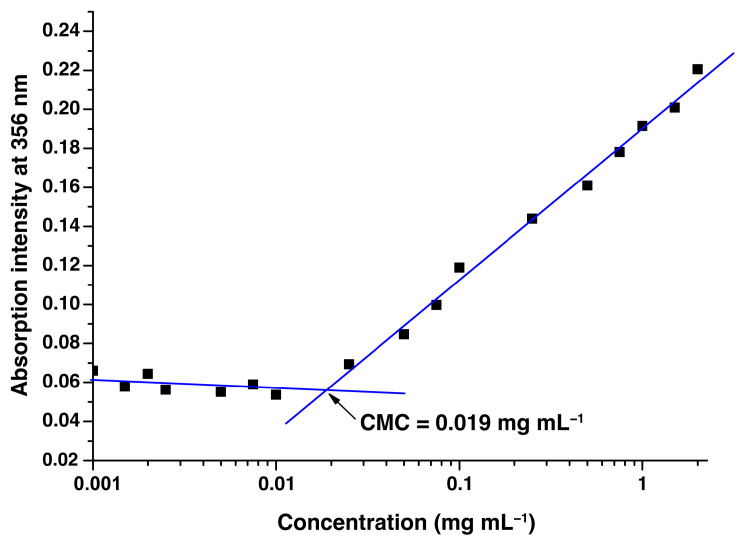
The effect of MPEG-*b*-PC block copolymer concentration on the absorption intensity of DPH at 356 nm in aqueous media.

An important parameter characterizing the supramolecular aggregates’ thermodynamic stability is the so-called critical micelle concentration (CMC), i.e., the concentration above which the aggregates are spontaneously formed. In order to estimate the CMC of the functional amphiphilic MPEG-*b*-PC diblock copolymer, a spectroscopic dye solubilization method was applied [43]. A series of copolymer aqueous solutions/dispersions with increasing concentrations in the presence of the hydrophobic dye DPH were prepared. The onset and the increasing of the dye solubilization into the micelles’ hydrophobic core were detected and followed by UV/Vis spectroscopy. The block copolymer CMC value was estimated from the curve presented in Figure 3.

The obtained value for the CMC of 0.019 mg mL^−1^ is in the micromolar range (1.95 × 10^−6^ M) and is an indication of enhanced thermodynamic stability of the block copolymer micelles formed. The results from the CMC evaluation are in good agreement with those obtained for amphiphilic block copolymers of similar compositions [42]. It is worth mentioning that all the further evaluations of the block copolymer micelles were performed at concentrations that were much higher than the estimated CMC value.

**Figure 4:**
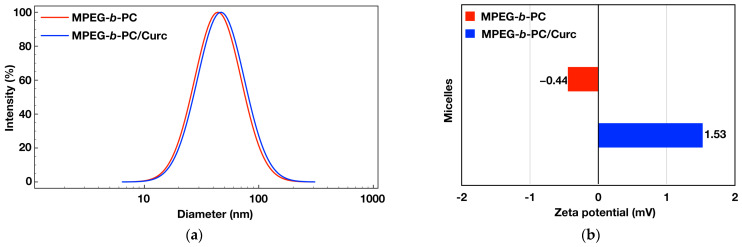
Size distributions by intensity (**a**) and zeta potentials (**b**) obtained from dynamic light scattering analyses of empty (MPEG-*b*-PC: d = 44 nm, PdI 0.245, ζ = −0.44 mV) and curcumin-loaded (MPEG-*b*-PC/Curc: d = 56 nm, PdI 0.208, ζ = 1.53 mV) block copolymer micelles.

The aqueous dispersions of the block copolymer were analyzed by dynamic light scattering. The results confirmed the formation of nanosized particles with monomodal and relatively narrow size distribution (Figure 4a, red curve). The detected average diameter of 44 nm is beneficial for the potential use of copolymer micelles as drug nanocarriers. It has been already demonstrated that average diameters of about 50 nm are optimal for nanoparticles intended for biomedical applications concerning their cellular uptake via the enhanced permeability and retention (EPR) effect [44]. At the same time, the size is large enough to prevent particles’ fast renal clearance and secure their longer systemic circulation. The particles’ size distribution curves by number are also presented (Figure S1). As expected, they revealed much smaller average diameters. The measured electrophoretic mobility and hence the calculated zeta potentials of −0.44 mV indicates, as expected, that the hydrophilic PEG surface of the micelles is neutral and would avoid the protein adsorption in potential in vivo applications (Figure 4b).

**Figure 5:**
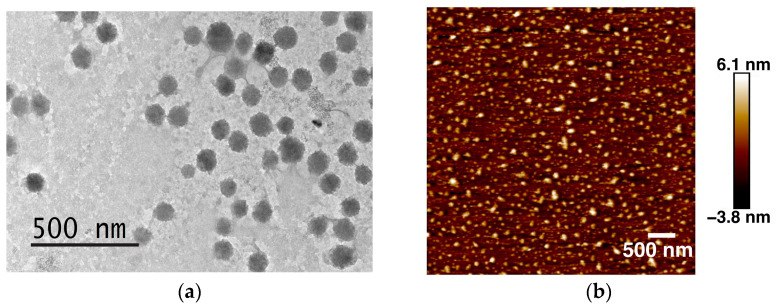
Morphology of MPEG-*b*-PC block copolymer micelles: (**a**) TEM image; (**b**) AFM image of individually dispersed particles.

The block copolymer micelles’ morphology was visualized via transmission electron microscopy (TEM) (Figure 5a) and atomic force microscopy (AFM) (Figure 5b).

The corresponding images obtained by both methods show the presence of spherical particles with average diameters that are slightly lower but still close to those detected by DLS measurements in a fully hydrated state. Most likely, the differences are due to the fact the sizes measured from both TEM and AFM analyses are referred to as collapsed micelles after solvent evaporation, whereas DLS determines the particles’ hydrodynamic diameter, which is sensitive to the shell swelling caused by water.

### 2.3. Drug Loading and In Vitro Release Studies

Following the positive results from the self-assembly and physico-chemical studies indicating the formation of stable nanosized spherical micelles from the MPEG-*b*-PC amphiphilic diblock copolymer, further efforts were focused on the evaluation of micelles’ ability to encapsulate hydrophobic drugs. Curcumin was chosen as a model drug due to its numerous biological activities but at the same time, it is characterized by poor water solubility and photo and chemical sensitivity. Moreover, the parameters concerning the drug loading and release evaluation could be relatively easily estimated due to the drug’s characteristic UV/Vis absorption spectrum. The model drug was loaded into the micelles during the process of their preparation. Thus, Curc was dissolved together with the copolymer at a 1:10 (*w*/*w*) ratio into a small, predetermined amount of organic solvent (acetone), and the solution was added dropwise to a larger aqueous volume. After the organic phase evaporation, the hydrophobic polymer blocks and the drug co-assembled into the core of the micelle. The final aqueous dispersion concentration was adjusted to 1 mg mL^−1^ and filtered through a 0.45 μm in order to remove the insoluble free drug. The drug-loaded micelles were recovered through lyophilization and destroyed by dissolving them into a predetermined amount of acetone. The drug loading efficiency (DLE) and the drug loading capacity (DLC) of the block copolymer nanocarriers were calculated using the data obtained from the respective UV/Vis absorption spectrum and applying Equations (3) and (4) shown in Section 3. The calculated values for the DLE and DLC of curcumin-loaded micelles were 62 wt% and 5.7 wt%, respectively.

The drug-loaded copolymer micelles (MPEG-*b*-PC/Curc) were also subjected to DLS analyses. The results obtained showed an approx. 10 nm increase in the average diameters for the drug-loaded micelles compared to the empty ones (Figure 4a). The increase in the size could be attributed to the hydrophobic core expansion as a result of drug solubilization. The size distribution was still monomodal and even narrower than the empty micelles. It might be speculated that the co-assembly process between the amphiphilic block copolymer and the hydrophobic drug is responsible for the formation of micelles with narrower size distribution. The particles’ average diameter increase after the drug loading is also clearly visible from the DLS size distribution curves by number (Figure S1). The zeta potential measurements on the drug-loaded micelles showed a slightly positive but still close to neutral surface charge (Figure 4b). Taking into account the standard deviations from the measurements, it might be concluded that there is no statistically significant difference in the surface charge between the empty and the drug-loaded micelles. This is an indication that the drug is loaded mainly into the core of the micelles. TEM and AFM analyses of the loaded block copolymer micelles confirmed that there was no change in particles’ morphology after drug encapsulation, just a slight increase in the average diameters (Figure S2). The characteristics of the block copolymer micelles before and after drug loading are presented in Table 2.

**Figure 6:**
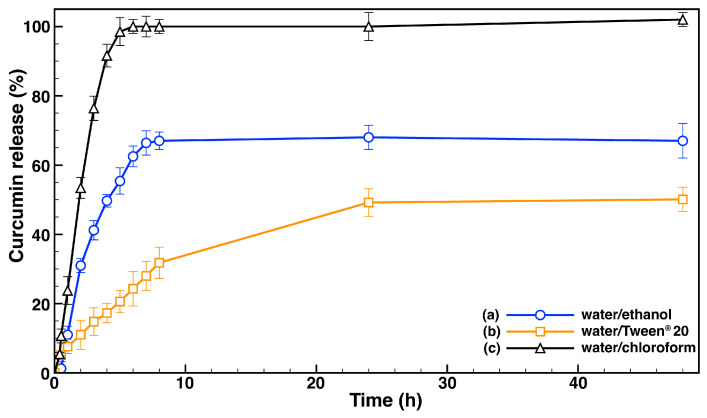
In vitro release profiles of curcumin from the block copolymer micelles at 37 °C in (**a**) distilled water containing 50% (*v*/*v*) ethanol; (**b**) distilled water containing 1% (*w*/*v*) Tween^®^ 20; (**c**) a biphasic (water/chloroform) system. Data are expressed as mean value ±SD, n = 3.

Drug release studies from the block copolymer micelles were performed to evaluate the impact of excipients on curcumin release. The in vitro drug release profiles from the block copolymer micelles were followed and evaluated in three different release media. The amount of the released curcumin at predetermined time intervals was quantified via UV/Vis spectroscopy. The cumulative percentages of drug release as a function of time are presented in Figure 6.

Initially, the curcumin release profiles were obtained using a dialysis membrane in order to separate the micelles dispersion from the aqueous release media containing drug solubilizing additives (Figure 6a,b). In cases when various amounts ranging from 2 to 20% (*v*/*v*) of ethanol were used to solubilize the released Curc, it was noticed that the drug remains trapped onto the membrane surface resulting in an incorrect release profile. Therefore, in order to prevent the adsorption and enhance the permeation of the drug across the dialysis membrane, the amount added to the release media ethanol was increased to 50% (*v*/*v*) (Figure 6a). However, the addition of such an amount of ethanol changed significantly the composition of the release media. In another experiment, 1% (*w*/*v*) Tween^®^ 20 was added to the aqueous release media, and no traces of curcumin retention onto the dialysis membrane were observed. The corresponding Curc release profile is presented in Figure 6b. Alternatively, the drug release profile was obtained by non-mixing with water organic solvent (chloroform) as a release media, thus avoiding the use of a dialysis membrane (Figure 6c). However, the so-called biphasic dissolution model is rarely used for drug release evaluation since there is a possibility of micelles’ dissolution at the water/organic solvent interface, leading to increased values for drug release [45]. All three profiles obtained under different conditions showed a burst drug release during the first hours of evaluation. In the case of the biphasic dissolution model, the whole amount of the encapsulated drug was released after 8 h, most likely due to a gradual micelle dissolution, as already discussed (Figure 6c). In the cases when aqueous release media were used, the first stage was followed by a sustained drug release (Figure 6a,b). The initial burst drug release followed first order-like kinetics might be due to the release of curcumin located on the periphery of the micelles’ core, close to the hydrophilic shell. The water/ethanol system showed fast initial drug release, reaching 68% of the released Curc for 48 h. The fast-release kinetics could be attributed to the micelles’ core swelling caused by the presence of 50% alcohol in the system (Figure 6a). On the contrary, just 28% of Curc was released from the micelles in aqueous media containing 1% Tween^®^ 20 after 8 h, followed by a much slower and sustained drug release reaching 50% for 48 h (Figure 6b). Since the water/Tween^®^ 20 system contains only a 1% solubilizing additive, it might be the most relevant for the Curc release evaluation. According to the results obtained, most of the drug would be safely preserved in the core of the micelle during the nanocarrier systemic circulation. However, the results from any in vitro drug release evaluation should be interpreted with caution since they are strongly dependent on the release media and the solubilizing additives used.

### 2.4. In Vitro Stability, Protein Adsorption, and Antioxidant Activity Evaluation

**Figure 7:**
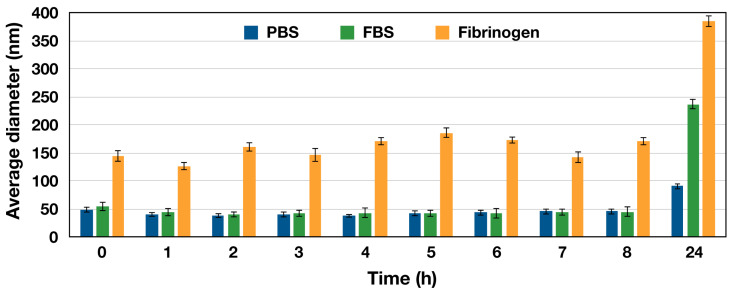
The stability of curcumin-loaded (MPEG-*b*-PC/Curc) micelles was assessed by DLS measurements in PBS (pH 7.4), FBS, and fibrinogen protein solutions after various time intervals of incubation at 37 °C. Data are expressed as mean value ±SD, n = 3.

The drug-loaded block copolymer micelle stability was evaluated in simulated post-intravenous injection blood conditions. The micelles were incubated in phosphate-buffered saline (PBS, pH 7.4), fetal bovine serum (FBS), and fibrinogen protein solutions at 37 °C and, at predetermined time intervals, the variations in their average diameters were detected by DLS measurements. The results showed that under simulated physiological conditions (PBS, 37 °C), the drug-loaded polymer micelles remained stable after 8 h of incubation with no significant change in average sizes (Figure 7).

A more noticeable increase in micelles’ diameters was detected after 24 h of incubation but was still below 200 nm. Similar evaluations were performed in two other biologically simulated proteins (FBS and fibrinogen) containing media. The potential protein adsorption estimation is important since if it takes place, it could lead to nanocarriers’ disassembly and untimely drug release during systemic circulation. The FBS solution did not affect block copolymer micelles’ sizes after 8 h of incubation (Figure 7). In the case when the drug-loaded micelles were incubated in the fibrinogen solution, somewhat bigger particles were formed from the initial time of mixing. Although some fluctuations in micelles’ diameters were detected after 8 h of incubation, they did not exceed 200 nm (Figure 7). After 24 h, both FBS and fibrinogen-incubated micelles significantly increased their diameters apparently due to protein adsorption. Nevertheless, it has been already shown that the maximum level of accumulated PEG coated micelles into the tumor tissue via the EPR effect, which is typically achieved within 6 h after their intravenous injection [46]. Consequently, the curcumin-loaded block copolymer micelles (MPEG-*b*-PC/Curc) demonstrate stability in biologically simulated media and might be suitable candidates for further in vitro and in vivo evaluations as safe nanocarriers of hydrophobic drugs.

**Figure 8:**
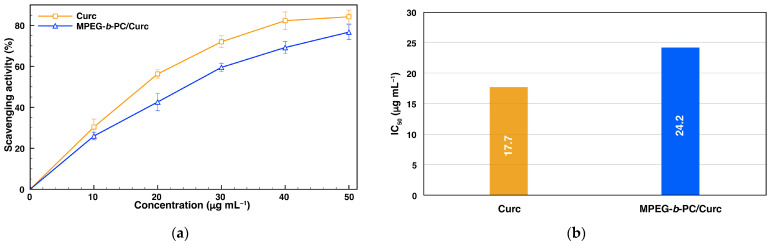
Determination of (**a**) DPPH• radical scavenging activity; (**b**) IC_50_ values of DPPH• radical scavenging activity of free curcumin (Curc) and curcumin-loaded block copolymer (MPEG-*b*-PC/Curc) micelles. Data on the scavenging activity vs. concentration curve are expressed as mean value ±SD, n = 3.

The in vitro antioxidant activity assay of the curcumin-loaded copolymer micelles aimed at determining whether the encapsulation of the drug into the nanosized carrier affects its biological properties. The DPPH• radical scavenging activity of free and micellar curcumin as a function of their increasing concentrations in ethanol/water mixture (1:1, *v*/*v*) is presented in Figure 8a. The obtained curves were used for the determination of the respective IC_50_ values. A higher free radical scavenging activity corresponds to a lower IC_50_ value (Figure 8b).

Thus, the free drug solubilized in the water/ethanol mixture exhibits better scavenging activity with IC_50_ = 17.7 μg mL^−1^ compared to the encapsulated curcumin. The IC_50_ value of the micellar curcumin was calculated to be 24.2 μg mL^−1^, indicating that a higher concentration from the micelles is needed to reduce activity by 50%. Nevertheless, the obtained value is still close to that of the free curcumin, which is an indication that the micellar drug’s antioxidant activity is preserved. Additionally, the scavenging activity of unloaded MPEG-*b*-PC block copolymer micelles was also performed. The results (not shown) revealed quite weak and concentration-independent activity, lacking IC_50_ value. Thus, the scavenging activity of the drug-loaded micelles is attributed solely to the curcumin itself with no contribution from the nanocarrier. It is worth noting that under in vivo conditions, a superior activity of the micellar curcumin is expected.

### 2.5. In Vitro Metabolic Activity and Morphology of MDCK II Cells Treated with Empty and Curcumin-Loaded Block Copolymer Micelles

**Figure 9:**
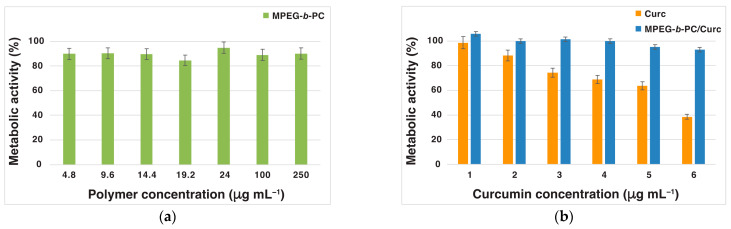
Metabolic activity via the MTT test of MDCK II cells after 6 h of incubation with different concentrations of (**a**) empty MPEG-*b*-PC block copolymer micelles; (**b**) free curcumin (Curc) and curcumin-loaded block copolymer micelles (MPEG-*b*-PC/Curc). Data are presented as percentages of untreated controls and are expressed as mean value ±SE, n = 3.

Cytotoxicity evaluations are widely used to demonstrate whether the newly synthesized compounds could affect the metabolic activity of cells and, if they could, cause cellular damage and/or cell death. The results of the MTT test performed on MDCK II cells treated with various formulations are presented in Figure 9. It is clearly visible that in the whole investigated wide concentration interval, the empty block copolymer micelles (MPEG-*b*-PC) exhibit minor cytotoxic potential (Figure 9a). The inhibition of cell metabolic activity was evaluated to be between 5 and 15% versus the untreated control. The established negligible cytotoxic effect of the empty block copolymer micelles is a favorable characteristic for their potential application as drug nanocarriers. As expected, curcumin exhibited a concentration-dependent cytotoxic effect on MDCK II cells (Figure 9b). On the other hand, no significant effect on the metabolic activity of cells treated with curcumin-loaded block copolymer micelles (MPEG-*b*-PC/Curc) was observed. This is an indication that the drug incorporation into the core of the non-toxic polymer micelles masks its toxicity on the evaluated normal cell line.

**Figure 10:**
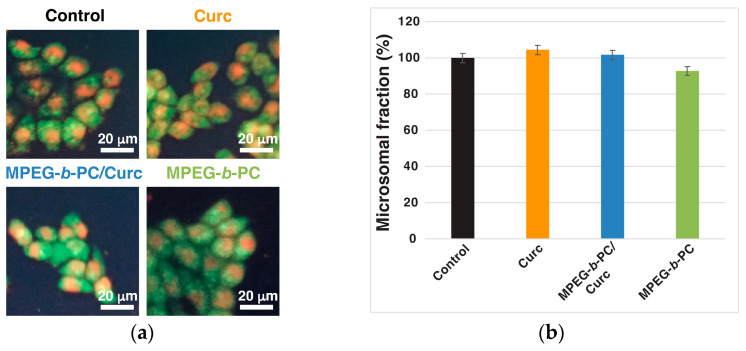
Acridine orange (AO) staining of MDCK II cells treated with curcumin (Curc), curcumin-loaded (MPEG-b-PC/Curc), and empty (MPEG-*b*-PC) block copolymer micelles. (**a**) Fluorescent microscope images of the control (untreated) and treated cells; (**b**) microsomal AO-stained fractions of the control and the treated cells.

Furthermore, no significant effect on the morphology of cells treated both with empty (MPEG-*b*-PC) and curcumin-loaded (MPEG-*b*-PC/Curc) block copolymer micelles compared to the untreated control cells was observed (Figure 10a). The difference between the acridine orange (AO, green to red)-stained microsomal fraction of treated and untreated (control) cells was found to be in the 5–7% range (Figure 10b). This negligible effect, as well as the low degree of cytotoxicity, suggests that both the empty and curcumin-loaded block copolymer micelles are biocompatible and do not affect the cellular metabolism of the evaluated normal cell line. The results obtained indicate that the functional copolymer micelles display protective properties and are promising candidates for further investigation as nanocarriers for biomedical applications.

Overall, the green synthetic path toward the functional amphiphilic block copolymer comprising biocompatible and biodegradable segments, its self-assembly into stable core-shell nanosized micelles followed by successful loading with a hydrophobic drug, and the performed in vitro evaluations altogether revealed the potential of the nanocarriers obtained for biomedical applications.

## 3. Materials and Methods

### 3.1. Materials and Reagents

All chemicals were purchased from Sigma-Aldrich (St. Louis, MO, USA). Methoxy-poly(ethylene glycol) (MPEG-5K, *M*_n_ = 5000 g mol^−1^) was freeze-dried from toluene. Tetrahydrofuran (THF, >99%) and *N,N*-dimethylformamide (DMF, ≥99.5%) were distilled from calcium hydride prior to use. Triethylamine (TEA, 99%) was distilled from potassium hydroxide. 2,2-Bis(hydroxymethyl)propionic acid (98%), propargyl bromide (80 wt% in toluene), ethyl chloroformate (97%), 4-dimethylaminopyridine (DMAP, >99%), 1,6-diphenyl-1,3,5-hexatriene (DPH, 98%), 2,2-diphenyl-1-picrylhydrazyl free radical (DPPH•), curcumin (Curc), potassium hydroxide (ACS reagent, ≥85%), Na_2_SO_4_ (≥99.0%), diethyl ether (for analysis), dichloromethane (DCM, ≥99.5%), and acetone (ACS reagent, ≥99.5%) were used as received.

5-Methyl-5-propargyloxycarbonyl-1,3-dioxane-2-one (MPC) was synthesized following a two-step already described procedure [47]. Briefly, 2,2-Bis(hydroxymethyl)propionic acid (3 g, 22.4 mmol) and potassium hydroxide (1.37 g, 24.4 mmol) were dissolved in 17 mL of DMF. After 1.5 h of stirring at 100 °C, propargyl bromide (4.1 mL, 80 wt% solution in toluene) was added dropwise, and the reaction was stirred at 70 °C for another 40 h. Finally, the resulting solids were filtered off and the filtrate was concentrated under a vacuum. The residue was dissolved in 5 mL of distilled water and extracted three times with 15 mL of DCM. The organic phase was dried over Na_2_SO_4_, and DCM was removed in a vacuum, yielding propargyl-2,2-bis(hydroxymethylpropionate) as a clear viscous liquid (2.95 g, 77%). In the next step, propargyl-2,2-bis(hydroxymethylpropionate) (3.15 g, 18.5 mmol) and ethyl chloroformate (4.02 g, 37 mmol) were dissolved in 50 mL of THF at 0 °C. After 30 min, TEA (4.5 g, 44 mmol) was added dropwise. Then, the mixture was stirred at room temperature for 24 h. Finally, the TEA.HCl salts were filtered off and the filtrate was concentrated in a vacuum to obtain a viscous liquid. The product was recrystallized from diethyl ether to give light brown crystals. Yield: 45%. ^1^H NMR (600 MHz, CDCl_3_, *δ*, ppm): δ 1.37 (s, 3H, -C*H*_3_), δ 2.54 (t, 1H, -CH_2_-C≡C*H*), δ 4.23–4.24 (d, 2H, -C*H*_2_-C(CH_3_)-CH_2_-), δ 4.72–4.74 (d, 2H, -C*H*_2_-C(CH_3_)-CH_2_-), and δ 4.79 (d, 2H, -C*H*_2_-C≡CH).

### 3.2. Solvent-Free Synthesis of Poly(ethylene glycol)-block-polycarbonate Functional Amphiphilic Copolymer (MPEG-b-PC)

MPEG-5K (1 g, 0.2 mmol) and MPC (0.99 g, 5.0 mmol) were freeze-dried from a toluene solution. Then, DMAP (49 mg, 0.4 mmol) was added, and the mixture was dried under a high vacuum for 30 min followed by flushing the flask with argon. The polymerization proceeded in bulk at 60 °C (oil bath) for 20 h. The crude product was extracted with 2-propanol, filtered, and dried to yield an MPEO-*b*-PC diblock copolymer. Yield: 1.8 g, 92%. ^1^H NMR (600 MHz, DMSO-d_6_, *δ*, ppm): 4.73 (s, OC*H*_2_C≡CH), 4.21–4.26 (m, OCH_2_C*H*_2_O(C=O) + OC(O)OC*H*_2_), 3.50 (s, *OCH*_2_*CH*_2_*O*), 3.23 (s, C*H*_3_O), 2.52 (s, CH_2_C≡C*H*), and 1.18 (s, *CH*_3_).

### 3.3. Characterization

^1^H NMR spectra were recorded in CDCl_3_ or DMSO-d_6_ on a Bruker Avance II+ 600 MHz instrument (Billerica, MA, USA). The average molar mass and dispersity of the polymers were determined by gel permeation chromatography (GPC) using a Shimadzu Nexera XR HPLC chromatograph equipped with a quaternary pump, degasser, automatic injector, column heater, UV/Vis (SPD-20A) detector, differential refractive index (RID-20A) detector, 10 μm PL gel mixed-B, and 5 μm PL gel 500 Å and 50 Å columns. The mobile phase was tetrahydrofuran (THF) with a flow rate of 1.0 mL min^−1^. The system was calibrated with polystyrene narrow molar mass standards. UV/Vis spectra were taken on a DU 800 Beckman Coulter spectrometer (Brea, CA, USA). Infrared spectra were obtained from an IRAffinity-1 Shimadzu Fourier Transform Infrared (FTIR) spectrophotometer (Kyoto, Japan) with a MIRacle attenuated total reflectance attachment. Transmission electron microscope (TEM) images were obtained using an HRTEM JEOL JEM-2100 (200 kV) instrument equipped with a CCD camera, GATAN Orius 832 SC1000, and GATAN Microscopy Suite Software. Atomic force microscope (AFM) images were taken on a Bruker NanoScope V9 instrument with a 1.00 Hz scan rate under ambient conditions. Observations were performed in ScanAsyst (Peak Force Tapping) mode. The average diameters and size distributions of the prepared micelles were determined by dynamic light scattering (DLS) using a NanoBrook Plus PALS instrument (Brookhaven Instruments), equipped with a 35 mW solid-state laser operating at λ = 660 nm and a scattering angle of 90°. The particles’ hydrodynamic diameters (*d_H_*) were determined according to the Stokes–Einstein equation:

*d_H_* = *kT*/(3*πηD*), (1)

where *k* is Boltzmann’s constant, *T* is the absolute temperature, *η* is the viscosity, and *D* is the diffusion coefficient.

The *ζ* potentials were calculated from the obtained electrophoretic mobility by the Smoluchowski equation:

*ζ* = 4*πημ*/*ε*, (2)

where *η* is the solvent viscosity, *μ* is the electrophoretic mobility, and *ε* is the dielectric constant of the solvent.

The size and zeta potential measurements were carried out in an automated mode in triplicate and recorded as averages of 3 and 20 runs, respectively. The polymer dispersions were passed through Millipore^®^ 0.45 μm pore-sized syringe filters prior to measurements.

### 3.4. Preparation of Micelles

The block copolymer micelles were prepared by the nanoprecipitation technique. The block copolymer was initially dissolved in acetone (10 mg mL^−1^). Then, 0.5 mL from the polymer solution was added dropwise to approx. 3 mL of ultrapure water (18.2 MΩ cm) under vigorous stirring. Finally, acetone was removed on a rotary evaporator, and the concentration of the micellar dispersions was adjusted to 1 or 2 mg mL^−1^ by the addition of ultrapure water.

### 3.5. Estimation of the Critical Micelle Concentration (CMC)

The dye solubilization method of Alexandridis et al. [43] was employed to determine the CMC of PEG-*b*-PC in water. Briefly, a series of block copolymer solutions with gradually increasing concentrations (from 0.001 to 2.0 mg mL^−1^) were prepared followed by the addition of 10 μL from a 0.4 mM solution in methanol of 1,6-diphenyl-1,3,5-hexatriene (DPH). After 18 h of incubation in the dark, the samples were subjected to UV spectroscopic analysis. The absorption intensity of DPH at λ_max_ = 356 nm was plotted as a function of block copolymer concentration. The CMC value for the amphiphilic diblock copolymer was determined as the intersection point of the two straight lines from the absorption intensity vs. concentration plots.

### 3.6. Drug Loading and In Vitro Drug Release Experiments

The preparation procedure of curcumin-loaded micelles was similar to the unloaded ones. Typically, curcumin was dissolved in acetone (1 mg mL^−1^), and 1 mL of solution was added to dissolve 10 mg of the block copolymer. Then, 0.5 mL from the copolymer/curcumin solution was added to 3 mL of water under vigorous stirring and after the acetone removal on a rotary evaporator, the concentration was adjusted to 1 mg mL^−1^ (micelles to curcumin ratio—10:1 *w*/*w*). The micellar dispersion was filtered (0.45 μm), lyophilized, and then resuspended in acetone for UV-vis spectroscopic analysis at a wavelength of 418 nm. A previously obtained value for the extinction coefficient, ε = 61,882 M^−1^ cm^−1^ (λ_max_ = 418 nm), of Curc in acetone was used to quantify the drug content encapsulated into the micelles [48]. The drug loading efficiency (DLE) and drug loading capacity (DLC) were calculated according to the following equations:

DLE (wt%) = (amount of curcumin in micelles/total initial amount of curcumin) × 100(3)

DLC (wt%) = (amount of curcumin in micelles/amount of the micelles) × 100(4)

The in vitro release of curcumin from the polymer micelles was studied in three different media. The micellar dispersions were placed into a dialysis membrane (MWCO = 50,000 Da) and immersed into 100 mL of the release media distilled water supplemented with Tween^®^ 20 (1% *w*/*v*) or ethanol (50% *v*/*v*). The studies were conducted at 37 °C under stirring of the release medium (100 rpm). At certain time intervals, samples were withdrawn from the buffered media, and the concentration of the released curcumin was determined by UV-vis spectroscopy (λ_max_ = 421 nm for water/Tween^®^ and λ_max_ = 424 nm for water/ethanol system). Alternatively, the in vitro release of Curc from the block copolymer micelles was studied using a non-mixing organic solvent (chloroform) as a release media. Typically, 4 mL of the aqueous Curc-loaded micelles’ dispersion (0.5 mg mL^−1^) were placed in a vial at 37 °C, followed by the addition of 2 mL of chloroform. At predetermined time intervals, 1 mL from the organic phase was withdrawn and subjected to UV/Vis spectroscopy (λ_max_ = 415 nm, ε = 53,703 M^−1^ cm^−1^) while the release medium volume was kept constant by the addition of an equal volume of fresh chloroform. The in vitro release experiments were run in triplicate.

### 3.7. In Vitro Stability and Protein Adsorption

The in vitro stability and protein adsorption of Curc-loaded nanoparticles were investigated in phosphate-buffered saline (PBS), fetal bovine serum (FBS), and fibrinogen solutions. Typically, 1 mL of PBS (pH 7.4), 10% (*v*/*v*) FBS, or 1 wt% fibrinogen was added to equal volumes of nanocarrier dispersions (1 mg mL^−1^). The dispersions were gently mixed at 37 °C, and the changes in the average particle diameters at different time intervals of incubation were followed by DLS measurements. The experiments were run in triplicate, and the results were expressed as mean value ±SD.

### 3.8. Antioxidant Activity Estimation via DPPH• Radical Scavenging Assay

The DPPH• scavenging activity of the curcumin-loaded micelles was determined using a previously described procedure with slight modifications [49]. Initially, 0.2 mM DPPH• solution was prepared in ethanol. For the analysis, 1 mL from block copolymer micelle dispersions in water prepared in a 10–50 mg mL^−1^ concentration range were mixed with 1 mL of DPPH• radical solution. The mixtures were left in the dark at room temperature for 30 min. The absorbance values of the mixtures were measured at a λ_max_ value of 517 nm by UV/Vis spectroscopy. The solution of DPPH• in ethanol/distilled water 1:1 (*v*/*v*) was used as a control. The DPPH• radical scavenging activity was calculated by the following equation:

% DPPH• radical scavenging activity = {(A0 − A1)/A0} × 100(5)

where A0 is the absorbance of the control and A1 is the absorbance of the sample.

The experiment was run in triplicate.

The calculated mean % of inhibition was plotted against the concentration, and from the graph, the IC_50_ value was calculated.

### 3.9. MTT Test

MDCK II cells were seeded in 96-well plates with initial concentrations of 1 × 10^5^ cells/mL in a DMEM medium, supplemented with 10% FBS (fetal bovine serum) and Penicillin/Streptomycin (Sigma-Aldrich, St. Louis, MO, USA). The cells were treated with curcumin and curcumin-loaded copolymer micelles (Curc concentrations between 1 and 6 µg mL^−1^) as well as empty copolymer micelles (concentrations between 4.9 and 250 µg mL^−1^) for 6h in cell media without FBS. MTT assays were performed as previously described [50].

### 3.10. Acridine Orange Staining

Acridine orange (AO) was previously used to visualize the microsomal fractions in eukaryotic cells after incubation with amphiphilic block copolymer micelles [51]. MDCK II cells were seeded at initial concentrations of 1 × 10^5^ cells/mL on coverslips. After 24 h, cells were treated with Curc (3 µg mL^−1^), empty polymer micelles (50 µg mL^−1^), and Curc-loaded polymer micelles (3 µg mL^−1^ curcumin) for 6 h in cell media without FBS. After incubation, the cells were washed with PBS and treated with AO (0.05% solution in PBS) for 5 min. Untreated cells incubated in a cell medium without FBS for 6h were used as a control. Images of the treated cells and controls were taken with a Nikon Eclipse system at 40× magnification using a 488 nm filter. The calculation of the total amount of the microsomal fraction was estimated using ImageJ free software, and the results were presented as a percentage of the control.

## 4. Conclusions

A solvent-free polymerization route for the synthesis of a well-defined polycarbonate-based functional amphiphilic diblock copolymer was established. Moreover, bulk polymerization was performed at a relatively low temperature. The block copolymer self-assembled in aqueous media into stable spherical micelles with a hydrophobic polycarbonate core and a hydrophilic PEG shell. The micelles were physico-chemically characterized and successfully loaded with the natural hydrophobic drug curcumin. Parameters such as drug loading efficiency and drug loading capacity were estimated. The results from the performed in vitro stability and protein adsorption evaluations of the drug-loaded micelles together with the high antioxidant activity of the encapsulated curcumin, as well as the results from the cell metabolic activity and cell morphology evaluations, are promising for their potential use in nanomedicine.

## Figures and Tables

**Table 1:** Molar mass characteristics of the MPEG-5K macroinitiator and the corresponding alkyne-functional amphiphilic diblock copolymer.

	Macroinitiator					Amphiphilic Diblock Copolymer				
Code	*DP_n_* ^a^	*M*_n_ ^a^ (g mol^−1^)	*M*_n_ ^b^ (g mol^−1^)	*Ð*_M_ ^b^	Code	t-*DP_n_* ^c^	*DP_n_* ^a^	*M*_n_ ^a^ (g mol^−1^)	*M*_n_ ^b^ (g mol^−1^)	*Ð*_M_ ^b^
MPEG-5K	114	5000	4500	1.03	MPEG-*b*-PC	25	24	9760	7350	1.24

^a^ Number average molar masses (*M*_n_) and degrees of polymerization (*DP_n_*), as determined by ^1^H NMR analyses. ^b^ Number average molar masses (*M*_n_) and molar mass dispersity (*Ð*_M_), as determined by GPC analyses. ^c^ Target degree of polymerization, t-*DP_n_* = [MPC]_0_/[OH]_0_, where [OH]_0_ is the active center concentration.

**Table 2:** Characteristics of block copolymer micelles before and after loading with curcumin.

Empty Micelles (MPEG-*b*-PC)			Curcumin Loaded Micelles (MPEG-*b*-PC/Curc)					
d ^a^ (nm)	PdI ^a^	ζ ^a^ (mV)	d ^a^ (nm)	PdI ^a^	ζ ^a^ (mV)	DLE ^b^ (wt%)	DLC ^b^ (wt%)	IC_50_^c^ (μg mL^−1^)
43.61 ± 0.42	0.245	−0.44 ± 0.75	55.88 ± 2.04	0.208	1.53 ± 1.64	62	5.7	24.20

^a^ Average micelle diameters (d), size distributions (PdI), and zeta potentials (ζ) obtained from DLS measurements. ^b^ Drug loading efficiency (DLE) and drug loading capacity (DLC) were determined spectroscopically. ^c^ The concentration of the antioxidant required to scavenge 50% DPPH• (IC_50_) was calculated from the inhibition vs. concentration curve.

## Data Availability

Not applicable.

## Supplementary Materials

The following supporting information can be downloaded at https://www.mdpi.com/article/10.3390/ijms241310588/s1.
