# Supplementary material for: Green Synthesis and the Evaluation of a Functional Amphiphilic Block Copolymer as a Micellar Curcumin Delivery System
Source: Int J Mol Sci. 2023 Jun 24;24(13):10588. doi: 10.3390/ijms241310588 (PMC10342159; doi:10.3390/ijms241310588)
Supplement: Supplementary file 1 [file ijms-24-10588-s001.zip › ijms-2451009-supplementary.pdf]

Supplementary Materials

Green Synthesis and the Evaluation of a Functional Amphiphilic Block Copolymer as a Micellar Curcumin Delivery System

Radostina Kalinova [1], Georgy Grancharov [1], Jordan Doumanov [2], Kirilka Mladenova [2], Svetla Petrova [2] and Ivaylo Dimitrov [1,*]

[1] *Institute of Polymers, Bulgarian Academy of Sciences, Akad. G. Bonchev St., bl. 103-A, 1113 Sofia, Bulgaria;*

[2] *Faculty of Biology, Sofia University "St. Kliment Ohridski", 8 Dragan Tzankov Blvd., 1164 Sofia, Bulgaria*

*Correspondence: dimitrov@polymer.bas.bg

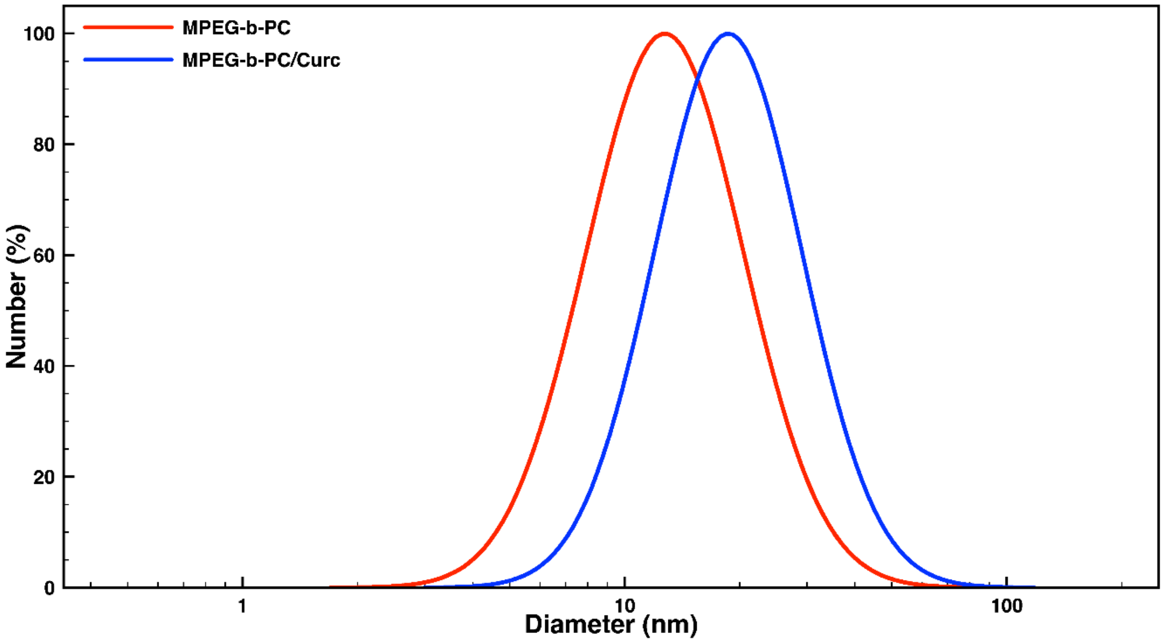

Figure S1. Size distributions by number obtained from dynamic light scattering analyses of empty (MPEG-*b*-PC) and curcumin-loaded (MPEG-*b*-PC/Curc) block copolymer micelles.

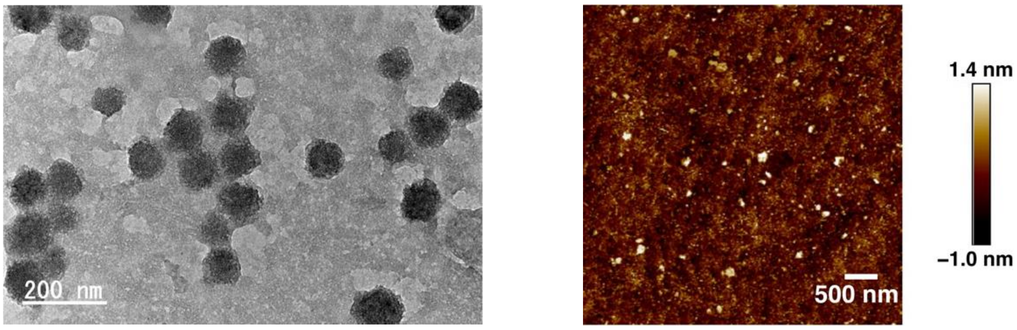

(**a**) (**b**)

Figure S2. Morphology of curcumin-loaded (MPEG-*b*-PC/Curc) block copolymer micelles: (**a**) TEM image; (**b**) AFM image of individually dispersed particles.
